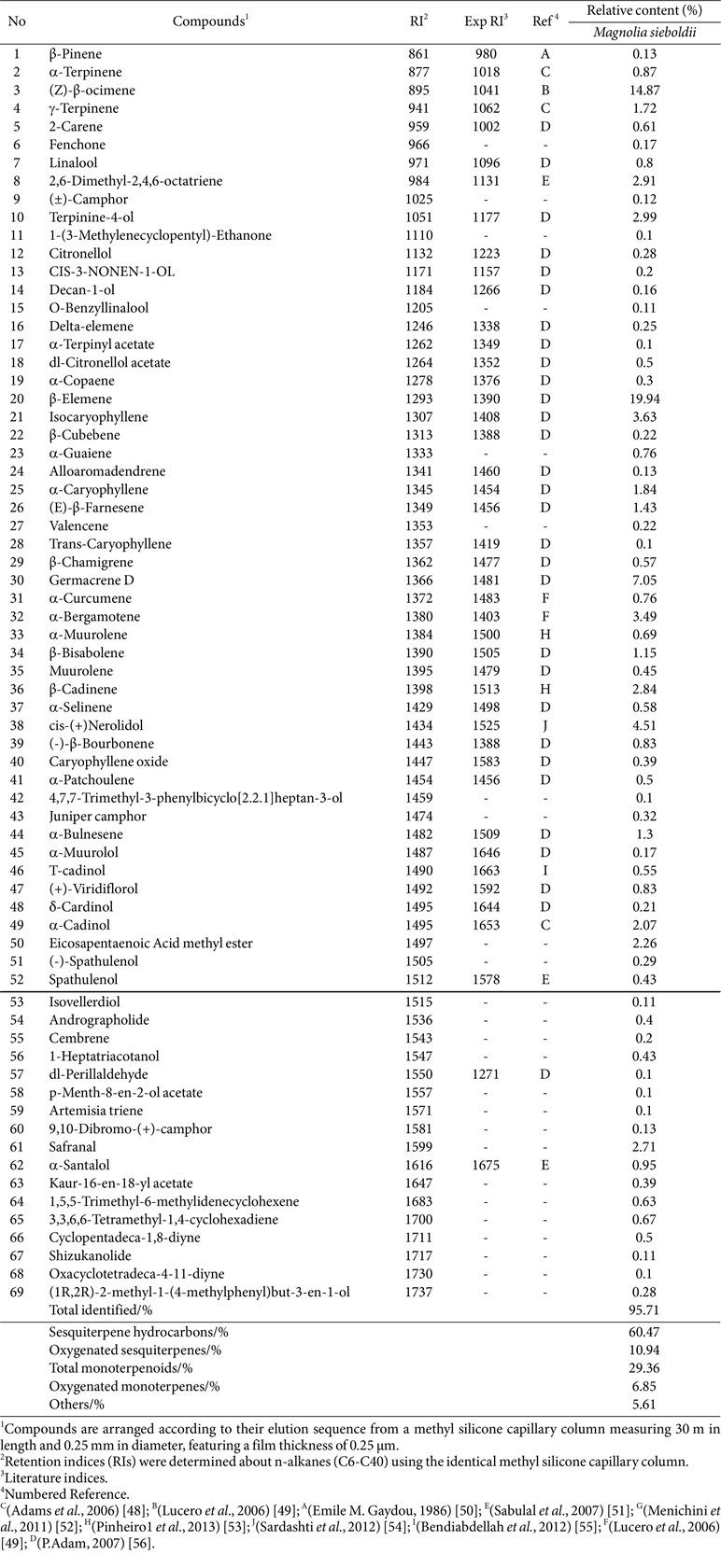# Research on the Improvement Effects and Mechanisms of *Magnolia sieboldii* Essential Oils on Insomnia in Mice

**DOI:** 10.4014/jmb.2502.02050

**Published:** 2025-05-26

**Authors:** Guofeng Shi, Shuanghe Wang, Shanshan Luo, Jiajing Ding, Zixuan Liang, Wenyu Cao, Xiaoyan Li, Yixi Zeng, Yanqing Ma, Lanyue Zhang, Hui Li

**Affiliations:** 1 School of Biomedical and Pharmaceutical Sciences, Guangdong University of Technology, Guangdong Provincial Key Laboratory of Plant Resources Biorefinery, Guangzhou 510006, P.R. China; 2 Department of Traditional Chinese Medicine, Institute of Guangdong Geriatric, Guangdong Provincial People's Hospital (Guangdong Academy of Medical Sciences), Southern Medical University Guangzhou 510080, P.R. China

**Keywords:** Essential oils, *Magnolia sieboldii*, insomnia, serotonin, γ-aminobutyric acid, glutamate decarboxylase

## Abstract

Insomnia is a common sleep disorder that is difficult to cure. *Magnolia sieboldii* essential oils (MSEOs) have been shown to have antidepressant effects, but there are few studies on treating insomnia. Therefore, this study aimed to investigate the therapeutic effects of MSEOs and to elucidate the molecular and neurophysiological mechanisms by which they alleviate insomnia. The main components of MSEOs extracted by steam distillation were analyzed by gas chromatography-mass spectrometry (GC-MS). To establish a p-chlorophenylalanine (PCPA)-induced insomnia model in mice, the levels of GAD65, GABAARα1, 5HT-2A, and 5HT-1A were detected by immunohistochemistry. The normal neurons in the mouse brain were counted by Nissl staining. RT-qPCR detected the relative mRNA expression levels of related genes in mice. A total of 69 components were identified by MSEOs, and the main components were β-elemene (19.94%), (Z)-β-ocimene (14.87%), and Germacrene D (7.05%). Different concentrations of MSEOs can successfully prolong the total sleep time and shorten the sleep latency of mice. GAD65, GABAARα1, 5HT-2A, and 5HT-1A levels still increased to varying degrees after treatment with different concentrations of MSEOs. Moreover, MSEOs could attenuate PCPA-induced neuronal death. At the same time, MSEOs enhanced the mRNA expression of 5HT-2A, GABAARα1, and GABAARγ2. MSEOs can extend sleep duration and reduce sleep latency in mice. MSEOs may demonstrate potential for treating insomnia by promoting neuronal proliferation in the brains of insomniac mice and upregulating the expression of GAD65, GABAARα1, 5HT-1A, and 5HT-2A proteins in various brain regions, potentially becoming an effective candidate for insomnia treatment.

## Introduction

Insomnia is marked by difficulties in starting to sleep, recurrent awakenings at night, and a diminished quality of life, a widespread epidemic [1]. The significance of adequate sleep is widely recognized, but due to increasing life pressures, the rate of insomnia continues to rise [2]. Numerous factors are intricately associated with the prevalence of insomnia, including gender, diet, constitution, environment, and age, although these possible influencing factors have not yet been definitively reported. Additionally, insomnia is recognized as a significant contributor to multiple systemic diseases. An epidemiological investigation has indicated that insomnia frequently coexists with depression and anxiety [3]. Meta-analyses indicate that those experiencing symptoms of disrupted sleep continuity or insomnia disorders are at a significantly higher risk of developing hypertension [4]. Benzodiazepines and non-benzodiazepines are currently effective drugs for the treatment of insomnia [5]. However, prolonged administration of these agents, especially among the elderly, is highly likely to lead to potential side effects, including cognitive impairment, dependence, and addiction [6, 7]. Consequently, herbal medicine, as a viable alternative therapy, offers the potential to treat insomnia with a reduced likelihood of adverse side effects.

The use of aromatherapy as a remedy for insomnia is globally acknowledged, encompassing the utilization of aromatic herbs within the realm of Traditional Chinese Medicine (TCM) [8, 9]. Aromatic herbs can activate brain functions and regulate the central nervous system. For example, *Lavandula angustifolia Mill*. and *Rosa rugosa* Thunb. had been used in clinical treatment [9, 10]. Their essential oils exhibit a limited incidence of adverse reactions and exert sedative-hypnotic properties [11]. Recent evidence highlights that plant extracts can enhance the operation of neurotransmitter systems and aid in sleep promotion. Existing research has demonstrated that *Magnolia sieboldii* essential oils (MSEOs) have sleep-promoting and antidepressant effects. However, the literature on this type of research is minimal, and the mechanism by which MSEOs promote sleep is not fully defined [12, 13].

The hypothalamus is a critical important brain region in mammals that regulates the sleep-wake cycle. The currently recognized central neurotransmitters involved in the regulation of the sleep-wake cycle include serotonin, norepinephrine, dopamine, acetylcholine, and γ-aminobutyric acid (GABA), among others. 5-Hydroxytryptamine (5-HT) plays a key role in regulating mood and sleep, and its deficiency is often associated with anxiety and depression; GABA, as the main inhibitory neurotransmitter in the central nervous system, helps to reduce neuronal excitability, promoting sleep onset and the maintenance of deep sleep [14]. GABA serves as an inhibitory neurotransmitter, contrasting with glutamate, which is excitatory. These two neurotransmitters are pivotal to the GABAergic system pathway, and any disruption in their equilibrium may lead to dysregulation of the sleep-wake cycle. Glutamate and GABA levels are modulated by glutamate decarboxylase (GAD) in synthesis. GAD facilitates the conversion of glutamate to GABA via decarboxylation, thus GAD's content and activity dictate the conversion efficiency from glutamate to GABA. [15, 16].

In sleep disorder research, the PCPA-induced insomnia model is a standard pharmacological tool for assessing sedative and hypnotic drug effects. This model's key mechanism involves PCPA inhibiting 5-HT synthesis, leading to a marked reduction in 5-HT levels in the peripheral and central nervous systems of animals, thus disturbing their circadian rhythms [17]. PCPA injection impairs the hippocampus, altering monoamine (*e.g.*, 5-HT, norepinephrine, dopamine) and amino acid (*e.g.*, GABA) neurotransmitter levels [18]. The PCPA model stands out for its brief cycle and pronounced insomnia, offering a valuable resource for evaluating insomnia treatments [19].

Therefore, the main objective of this study is to analyze the primary components of MSEOs, their efficacy in the treatment of insomnia, and the mechanisms of action in terms of neurotransmitters and neurophysiology. In this investigation, we employed a PCPA-induced insomnia model to assess the sleep-enhancing properties of MSEOs an analysis will be conducted focusing on the expression levels of 5-HT, GAD65, and GABA, along with normal neuronal levels in brain tissue. The objective of this research is to offer a scientific basis for further understanding the mechanism of MSEOs in the management of insomnia, as well as to promote more research into their clinical application, hoping to provide more solid support for clinicians' treatment plans.

## Methods and Materials

### Plant Essential Oils

Dried leaves of *Magnolia sieboldii* (5 kg) were collected from Jangsu Province (China). All plant samples were identified by Professor Nian Liu (Zhongkai University of Agriculture and Engineering, China). As voucher specimens, part of the collected sample has been deposited for safekeeping at the Institute of Natural Medicine and Green Chemistry (Guangdong University of Technology) (Table 1). Use a pulverizer to grind the leaves of *Magnolia sieboldii* into fine powder. Mix them with a little water in a beaker to form lumps. Put them in a microwave oven and heat for 2 min. Mix them with water in a ratio of 1:8 in an efficient essential oils distillation equipment (model: TX05-02, China), and collect the essential oils for 4 consecutive h. Add anhydrous sodium sulfate to remove the water and store it at 4°C for experiments. The yield of essential oils was determined using the subsequent formula:

Essential oils yield (%) = obtained essential oils (g) / dried raw material (g) × 100%

### Conditions for GC-MS Determination

Essential oils were detected by a DSQ-II Ultra gas chromatography-mass spectrometry (GC-MS) (Thermo Fisher Scientific, USA), which was equipped with a DB-5MS capillary gas chromatographic column (0.25 m 0.33 μm) (Agilent, USA). Temperature program: 100°C (10 min), 3°C/min, 250°C. The vaporization temperature is 250°C. The volume of the injection was 1.0 μl. The helium flow was 1.0 ml/min. Injection method was shunt. The split ratio was 20:1. The bar of mass spectrometry: ion source was electron blast; the temperature was 200°C; eneray was 70 eV, scanning range was m/z 30~550 amu. Retention indices (RIs) were determined using n-butane standard samples according to the Kovats method. The identification of metabolites was based on the NIST library and Wiley library, supplemented by an in-house standard library. The identification criteria included a mass spectral similarity score (>80%) and retention index (RI) matching. These rigorous criteria ensure the accuracy and reliability of metabolite identification in our analysis. To ensure the reproducibility of our GC-MS analysis, we rigorously evaluated the method by analyzing three independent batches prepared and analyzed on different dates, incorporating quality control samples (blanks, internal standards, and reference materials) in each batch. Statistical evaluation confirmed reproducibility, with relative standard deviation (RSD) values within acceptable limits (<15%) across batches and replicates. These measures collectively ensure the reliability and reproducibility of our GC-MS analysis.

### PCPA-Induced Insomnia

**Fig. 1:**
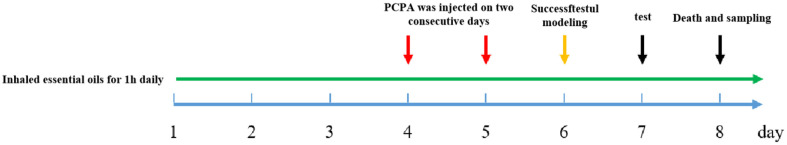
Flow chart of the experiment.

24 healthy adult male KM mice, SPF grade, aged 6-8 weeks were from Southern Medical University (license number: SCXK (Guangdong)2016-0041). All the animal experiments were performed under the Guidelines for Care and Use of Laboratory Animals of Guangdong University of Technology, and experiments were approved by the Animal Ethics Committee of Guangdong University of Technology. Experimental mice were adaptively fed for a week under conditions of 24 ± 1°C temperature and a 12-h light/dark cycle. A total of 24 male Kunming mice were stochastically divided into six groups, including the MSEOs-L group (25 mg/kg), MSEOs-M group (50 mg/kg), MSEOs-H group (100 mg/kg), control group, PCPA group, and diazepam (DZP) group (0.2 mg/ml). Filter paper is cut into the same size and soaked in different concentrations of essential oil solutions. Then, filter paper was placed in four corners of the cage so that each mouse could sniff it for an hour. The experimental procedure is shown in Fig. 1. The cage housing the control group mice was equipped with filter paper that had been soaked in a normal saline solution with 1% Tween 80 for a duration of 1 h. Four days into the animal study, except for the control group of mice injected with saline containing 1% Tween 80 pH 7.8, the other groups of mice were administered with PCPA at a dosage of 0.1 ml per 10 grams of the animal's body mass for two days. Behavior observation was carried out in mice after the injection. The mice exhibited continuous activity throughout the day, displaying heightened levels of excitability and irritability. Additionally, they experienced a disruption in their circadian rhythm, along with elevated water and food consumption, while their sleep duration was markedly reduced. The group injected with PCPA was significantly different from the control group.

### Pentobarbital-Induced Sleep Test

**Fig. 2:**
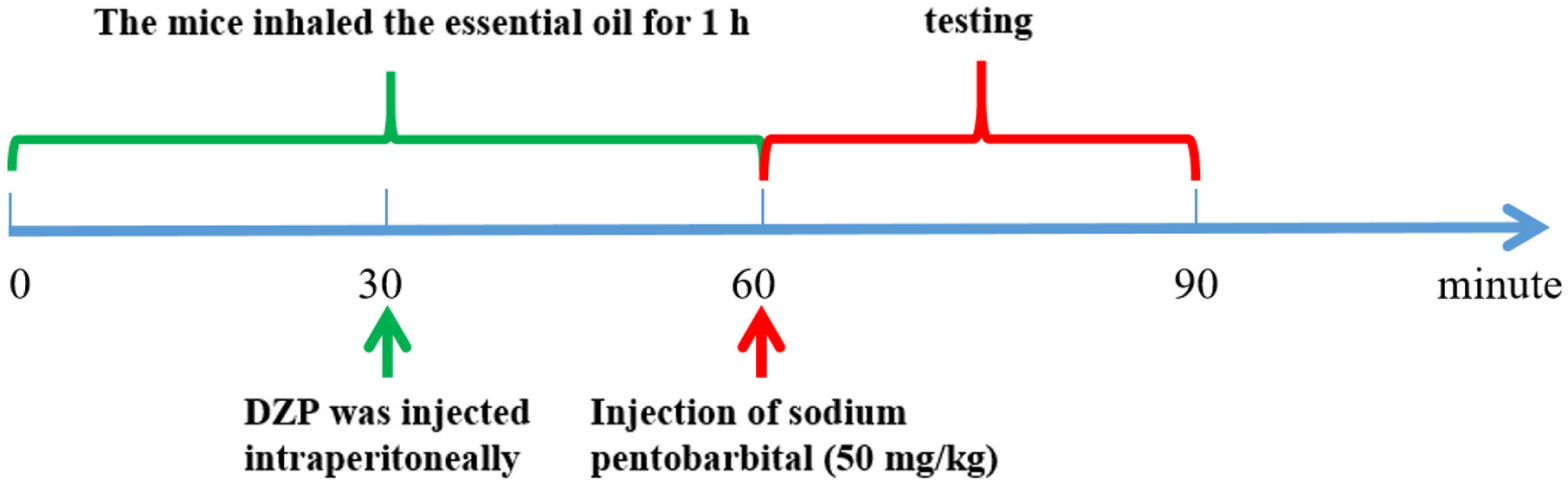
Test flow chart.

Fig. 2 outlines the test protocol; sodium pentobarbital was injected intraperitoneally into mice at 50 mg/kg, 30 min after the last dose, to induce sleep. Observations included the number of turnovers and sleep status, time to sleep onset (TS), time of drug administration (TR), latency to sleep, and duration of wakefulness after sleep (TW).

Latency = TS - TR, Total Sleep Time = TW- TS

### BCA Assay

Following the above experiment, the mice were subjected to euthanasia, and blood samples were obtained through the excision of the ocular globes. Blood samples were subjected to centrifugation at a rotational speed of 2,500 rpm for a period of 5 min at 4°C in order to isolate the serum. Protein levels in serum were measured by a bicinchoninic acid (BCA) protein assay kit (China).

### ELISA Assay

The level of 5-HT in the hypothalamus of mice was measured using an ELISA assay kit (China). After the experiment, the mice were euthanized, and the hypothalamic tissue was quickly removed and then rinsed with cold saline to eliminate residual blood. Hypothalamic tissues were washed with phosphate-buffered saline (PBS), homogenized, and centrifuged, and the supernatant was collected. The procedure was carried out in line with the instruction manual provided in the ELISA kit, and absorbance was assessed at a wavelength of 450 nm, a standard curve was established to calculate the concentration of each detection index.

### Immunohistochemistry Assay

Post-euthanasia, mouse brains were excised on an ice surface, then rinsed with saline and dried with filter paper to remove residual blood. Brains were fixed in 4% paraformaldehyde for 24 h, followed by embedding in paraffin and sectioning at 4 μm using a microtome (CUT 5062, China). The slides were dried for 2 h at 60°C. Afterward, the specimens were soaked in absolute ethanol solutions I and II for 10 min each, this was followed by 5-min immersions in 95%, 90%, 85%, and 70% ethanol. Afterward, then wash the slices with PBS and immerse them in a buffer solution of sodium citrate adjusted to a pH of 6.0 under elevated temperatures for antigen repair. Subsequently, mouse brain tissue sections underwent treatment with 3% H_2_O_2_ for 25 min under dark conditions so that endogenous peroxide was blocked, tissues were blocked using 1.5% normal goat serum. Tissue sections were incubated with a 15,000-fold diluted rabbit anti-serotonin antibody (Incstar, USA) at 37°C for 2 h. Following this, the samples were processed with a 12,500-fold diluted rabbit anti-serotonin transporter antibody (Incstar) for 15 min, and then a 5,000-fold diluted rabbit anti-tyrosine hydroxylase antibody (Pel-Freez Biologicals) for an additional 15 min. Immunoreactivity was visualized using 3,3'-diaminobenzidine (DAB) for the color reaction.

### Nissl Staining Assay

The embedded paraffin tissues were sliced at 5 μm and soaked in xylene I and xylene II for 10 min each, subsequently in 95%, 90%, 85%, and 70% ethanol with each stage lasting 5 min. The sections were subjected to staining with a 0.1% solution of cresyl violet for 20 min, followed by rinsing with distilled water. Dehydration and clearing of paraffin sections were performed as follows: First, paraffin-embedded tissue sections were subjected to primary dehydration in 70% ethanol solution for 2 min. Subsequently, the sections were transferred to 80%ethanol solution for intermediate dehydration, again maintained for 2 min. Further, the sections were subjected to advanced dehydration in 95% ethanol solution and the treatment time was also 2 min. Immediately following, complete dehydration with absolute ethanol, and the sections were immersed in 100% ethanol for 5 min each time for a total of two sessions. Then, after dewaxing and clearing, the sections were immersed in xylene for 10 min each time to complete two clearing steps. Finally, the paraffin sections were sealed with resin.

### RT-qPCR

Upon conclusion of the two-week treatment regimen, the hypothalamic and hippocampal regions were swiftly excised from the mouse skulls. These excised tissues were subjected to a delicate rinsing process with chilled physiological saline before being flash-frozen on dry ice. Following this, they were preserved at a temperature of -80°C. A sample weighing 50 milligrams from either the hypothalamus or hippocampus was processed in Trizol for a comprehensive homogenization to isolate total RNA. The synthesis of cDNA was carried out by the protocols provided with the cDNA synthesis kits. After this, the produced cDNA was subjected to amplification using primer sequences as detailed in Table 2. Calculation method: β-actin was used as the internal reference gene for normalization, and the 2^−ΔΔCT^ method was applied to calculate the gene expression level.

### Statistical Analysis

To ensure the reliability and statistical power of the experimental results, we designed the study with 4 independent replicates based on preliminary outcomes, expected effect sizes, and field standards. Through sample size calculation, referencing similar research methodologies, and strict adherence to experimental protocols, we are confident that this level of replication is sufficient to achieve a power level of at least 0.8, thereby ensuring data adequacy and the validity of statistical tests [20, 21]. A comprehensive one-way analysis of variance was conducted on the entire dataset with the aid of GraphPad Prism version 8.0.2 (developed by GraphPad Software, USA). After this, a Tukey-Kramer post-hoc test was applied to ascertain any statistically significant disparities between the control and experimental cohorts, with a significance threshold set at *p* < 0.05 or *p* < 0.01. To conduct a robust statistical power analysis and to establish the appropriate sample size, R version 4.2.3 software (provided by the R Foundation for Statistical Computing, based in Austria) was utilized, in conjunction with the 'pwr' add-on. The findings are presented as mean values accompanied by standard errors, and the level of confidence for these results is set at 95%. This data was meticulously gathered through a minimum of three separate and autonomous experimental replicates.

## Results

### Essential Oils Component Analysis

The GC-MS analysis yielded results (Table 3) identifying 69 compounds, which comprised 95.71% of the overall essential oils composition. There were 24 Sesquiterpene hydrocarbonszn (60.47%), 13 Oxygenated sesquiterpenes (10.94%), 11 Total monoterpenoids (29.36%), and Oxygenated monoterpenes 6 (6.85%) and 15 other substances (5.61%). The content of β-elemene (19.94%) was the highest. Followed by (Z)-β-ocimene (14.87%), Germacrene D (7.05%), cis-(+)Nerolidol (4.51%), isocaryophyllene (3.63%), etc. β-elemene has ameliorative effects on inflammation in the CNS[22] and is found as a major component of many plant essential oils that promote hypnosis [23, 24]. Several studies have shown that Nerolidol is an important component capable of inducing sedation in mice [25], as indicated by decreased motility and prolonged sleep duration induced by pentobarbital [23]. Nerolidol has a protective effect against neuroinflammation [26] and the anti-inflammatory activity is associated with the gabaergic system [27]. Nerolidol enhances neuronal effects by stimulating the activation of 5-HT receptors, particularly the 5HT-1A and 5HT-4 subtypes [28]. Isocaryophyllene has good anticancer activity [29], but its effect on the nervous system has not been reported. The effect of MSEOs on improving sleep disorders may be related to the effects of β-elemene and Nerolidol on the nerve center, as well as the results of the interaction with other components.

### Impact of MSEOs on Sleep Duration and Latency to Sleep Onset in Insomniac Mice

**Fig. 3:**
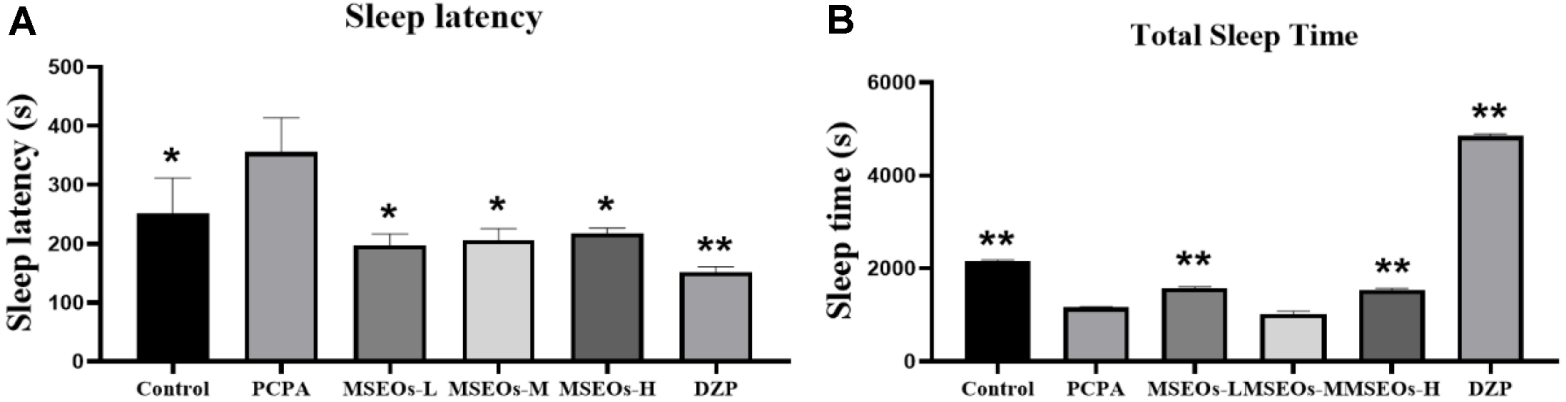
Impact of MSEOs on latency to sleep onset and total sleep duration in mice with PCPA-induced insomnia. (**A**) Comparison of sleep latency between different groups of mice. (**B**) Comparison of sleep duration between different groups. (*p < 0.05 and **p < 0.01 highlight notable distinctions from the PCPA-treated model group. MSEOs-L, MSEOs-M, and MSEOs-H represent dosages of 25 mg/kg, 50 mg/kg, and 100 mg/kg, respectively.) Data showed mean ± SD (*n* = 4).

The experimental results are shown in Fig. 3. The sleep latency of PCPA group mice was significantly longer than that of the control group (*P* < 0.05) (Fig. 3A). Additionally, the total sleep duration of the PCPA group mice was markedly shorter than that of the control group (*P* < 0.01) (Fig. 3B). This indicates that the insomnia mouse model has been successfully established. After treatment with MSEOs, compared to the insomnia model group, the sleep latency of mice treated with three concentrations of MSEOs was significantly reduced (*P* < 0.05)(Fig. 3A). Moreover, the sleep duration of mice in the MSEOs-L and MSEOs-H treatment groups was significantly increased compared to the PCPA group (*P* < 0.01) (Fig. 3B). In summary treatment with MSEOs can effectively enhance sleep quality in mice with insomnia caused by PCPA.

### Neuroprotective Effects of MSEOs on PCPA-Induced Insomnia Mice

**Fig. 4:**
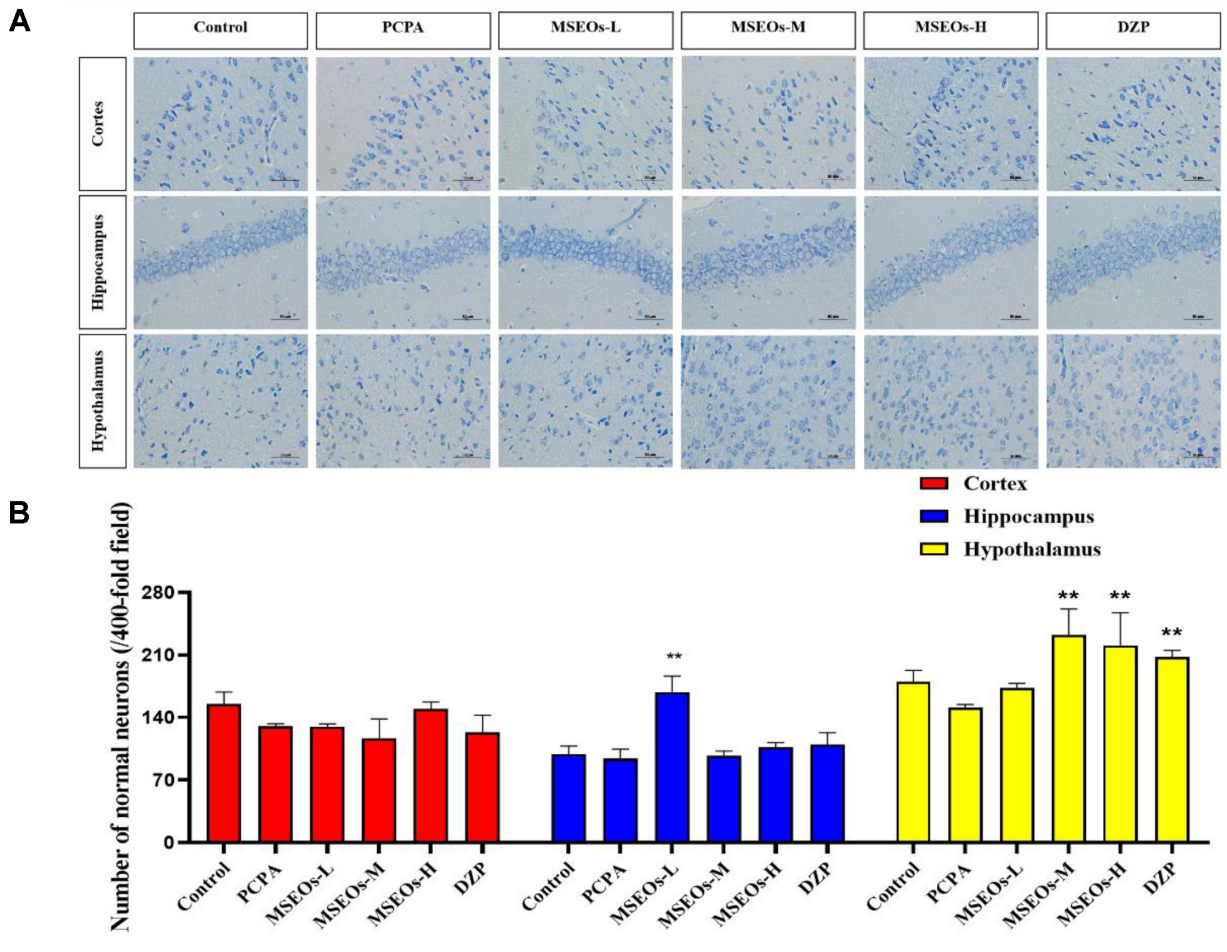
A comparative analysis of neuronal quantities across various brain tissues. (**A**) Nissl's staining. (**B**) Quantity of uninjured neurons in each region of mouse brain tissue. (**p < 0.01 highlight notable distinctions from the PCPAtreated model group. MSEOs-L, MSEOs-M, and MSEOs-H represent dosages of 25 mg/kg, 50 mg/kg, and 100 mg/kg, respectively.) Data showed mean ± SD (*n* = 4).

Insomnia is closely associated with brain neuron damage. Hippocampal neuron damage or atrophy is detrimental to rapid eye movement sleep [30]. Moreover, more severe insomnia may be caused by hypothalamic injury[31]. Compared to the control group statistical analysis of the count of neurons in the hypothalamus, hippocampus, and cerebral cortex revealed that the PCPA group had fewer uninjured neurons located within the cerebral cortex, hypothalamus, and hippocampus. However, after administration of MSEOs, the count of normal neurons located within the hippocampus, cerebral cortex, and hypothalamus increased, especially with MSEOs-M and MSEOs-H in the hypothalamus and MSEOs-L in the hippocampus showing the most pronounced effects (*p* < 0.01) (Fig. 4B). This indicates that MSEOs have a protective effect on neurons, which may be related to their function in treating sleep disorders.

### Expression of 5HT-2A and 5HT-1A

Serotonin, also widely recognized as 5-HT, is a classical neurotransmitter in the central nervous system that significantly influences the regulation of sleep and wakefulness. Serotonin is acknowledged as one of the key mechanisms contributing to insomnia [32]. Its two important subtypes are 5HT-1A and 5HT-2A receptors, respectively. The 5HT-2A and 5HT-1A proteins are extensively distributed throughout the brain, and play a role in the modulation of sleep [33, 34], anxiety, and emotions [35]. Low-dose 5HT-1A receptor agonists are known to boost both deep and light sleep phases [36]. PCPA can cause insomnia by inhibiting presynaptic 5HT-1A autoreceptors and reducing 5-HT levels [37].

**Fig. 5:**
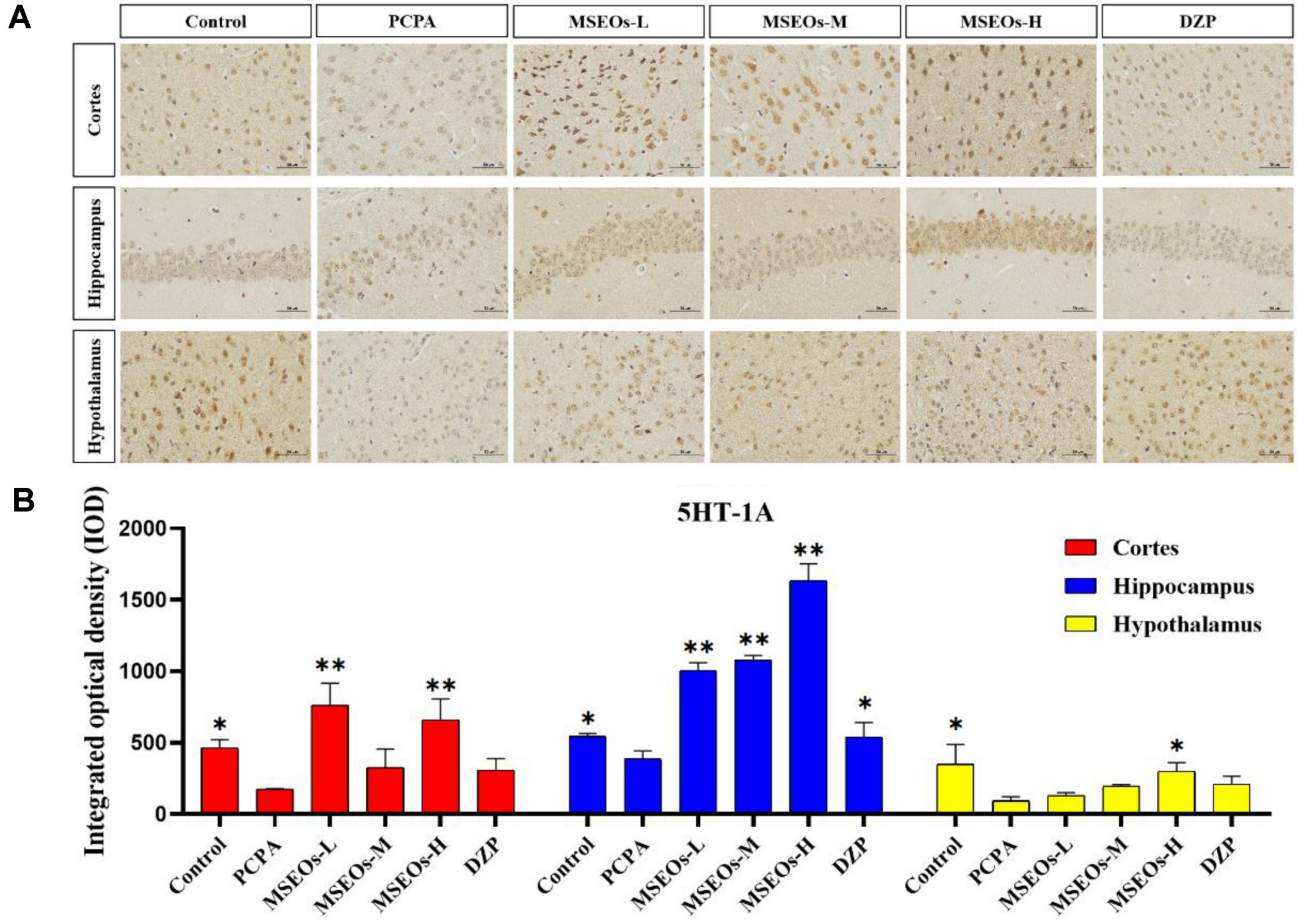
Expression of 5HT-1A protein. (**A**) Immunohistochemical staining images of 5HT-1A protein; (**B**) Quantitative assessment of 5HT-1A expression in various mouse brain regions. (*p < 0.05 and **p < 0.01 highlight notable distinctions from the PCPA-treated model group. MSEOs-L, MSEOs-M, and MSEOs-H represent dosages of 25 mg/kg, 50 mg/kg, and 100 mg/kg, respectively.) Data showed mean ± SD (*n* = 4).

**Fig. 6:**
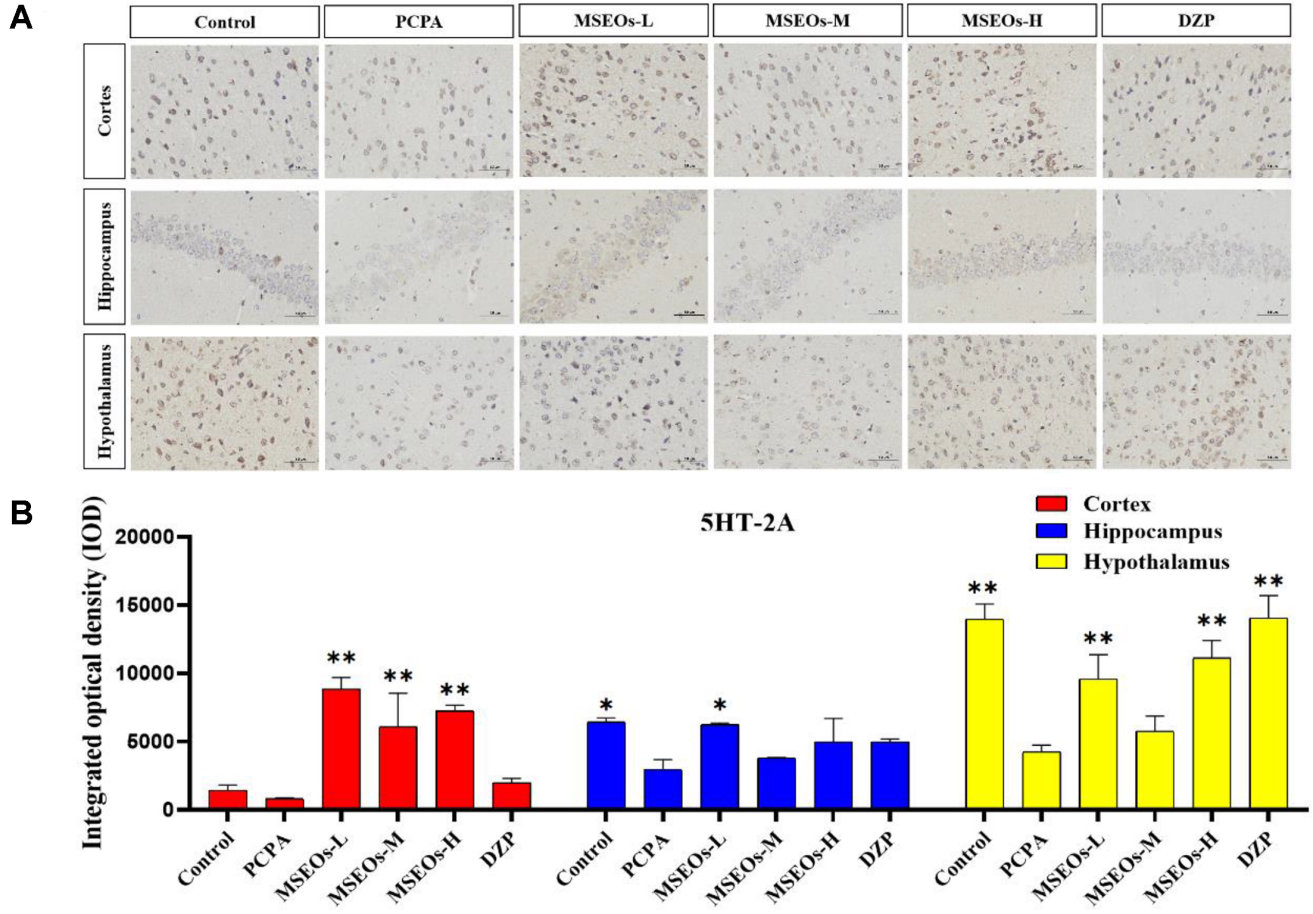
Expression of 5HT-2A protein. (**A**) Immunohistochemical staining images of 5HT-2A protein; (**B**) Quantitative assessment of 5HT-2A expression in various mouse brain regions. (*p < 0.05 and **p < 0.01and *p < 0.05 highlight notable distinctions from the PCPA-induced model group. MSEOs-L, MSEOs-M, and MSEOs-H represent dosages of 25mg/kg, 50 mg/kg, and 100 mg/kg, respectively.) Data showed mean ± SD (*n* = 4).

The test results show (Fig. 5) that compared to the normal control group the 5HT-1A expression in mice's cortex, hippocampus, and hypothalamus within the PCPA group is notably lower (*P* < 0.05) (Fig. 5B), and in the cortex, hippocampus, and hypothalamus, the expression level of 5HT-2A was also decreased (*P* < 0.01) (Fig. 6B). Compared to the insomnia model group, MSEOs treatment resulted in increased levels of 5HT-1A in the cerebral cortex, hypothalamus, and hippocampus of mice across three dosage groups, with the MSEOs-H group showing the most favorable results in the hippocampus and hypothalamus (*p* < 0.05) (Fig. 5B). The expression levels of 5HT-2A in the cortex, hypothalamus, and hippocampus of mice in three dosage groups also increased, with MSEOs-L showing better effects in the cortex and hippocampus (*p* < 0.05) (Fig. 6B). It can be seen that treatment with MSEOs effectively inhibited the decrease in 5HT-2A and 5HT-1A levels in mice exhibiting PCPA-induced insomnia, maintaining the levels of 5HT-2A and 5HT-1A in the mice at normal levels.

### Effect of MSEOs on GABAARα1 Levels in Mice with Insomnia

GABA, a pivotal inhibitory neurotransmitter, significantly curbs the activity of the majority of neurons in the suprachiasmatic nucleus [38]; its inhibitory actions are closely tied to animal circadian rhythms [39]. GABA is commonly used as an important indicator for evaluating central nervous system (CNS) function and is also a common metric in insomnia research [40], with the A receptor subtype GABAARα1 being the most prevalent [41]. Studies have shown that the expression levels of GABAARα1 and GABA were significantly decreased in the PCPA-induced insomnia mouse model [42]. To ascertain if MSEOs can enhance sleep quality by regulating the expression of GABAARα1 in the subject animals, we quantitatively assessed the GABAARα1 levels in mouse brain tissue.

**Fig. 7:**
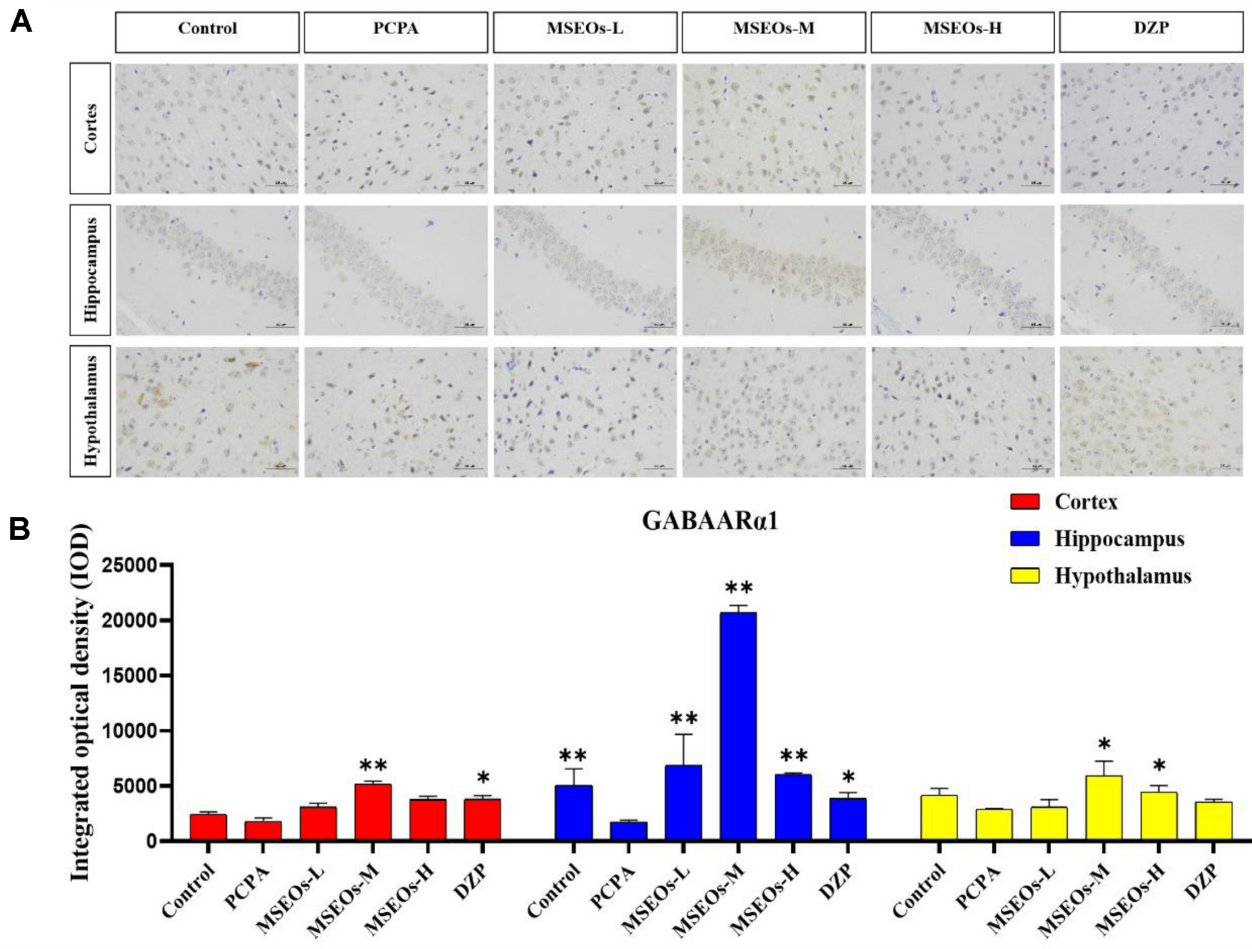
Expression of GABAARα1 protein. (**A**) Images of GABAARα1 protein staining in various regions of mouse brain tissue; (**B**) Quantitative assessment of GABAARα1 proteins in various brain tissues. (*p < 0.05 and **p < 0.01 highlight notable distinctions from the PCPA-induced model group. MSEOs-L, MSEOs-M, and MSEOs-H represent dosages of 25 mg/kg, 50 mg/kg, and 100 mg/kg, respectively.) Data showed mean ± SD (*n* = 4).

Compared with the control group, the expression of GABAARα1 in the cortex, hippocampus, and hypothalamus of the insomnia model group was decreased (Fig. 7). It can be seen that PCPA can cause a decrease in the GABAARα1 content in mice exhibiting insomnia. However, after intervention with MSEOs, the GABAARα1 levels in brain tissues of MSEOs mice were higher than those of PCPA mice, especially in the MSEOs-M group (*p* < 0.01). This indicates that MSEOs can inhibit the decrease in GABAARα1 levels in PCPA-induced sleep disorders in mice, maintaining the GABAARα1 content in the mice at normal levels.

### Impact of MSEOs on GAD65 Protein Levels in the Brain of Mice Subjected to Sleep Deprivation

GABA in the brain mainly catalyzes glutamate synthesis through the GAD enzyme [16]. Two glutamate decarboxylase isoforms exist: GAD67 and GAD65 [43]. Studies indicate that GAD65 is responsive to GABA fluctuations, predominantly located in the cytoplasm of GABAergic neurons, and primarily drives GABA synthesis and release in the brain [44]. Previous research has indicated a decrease in GAD65 levels within the cerebral tissue of mice experiencing PCPA-induced sleep disorder treatment [45]. Therefore, the anti-insomnia effect of MSEOs could be determined by GAD65 protein expression in mouse brain tissue.

**Fig. 8:**
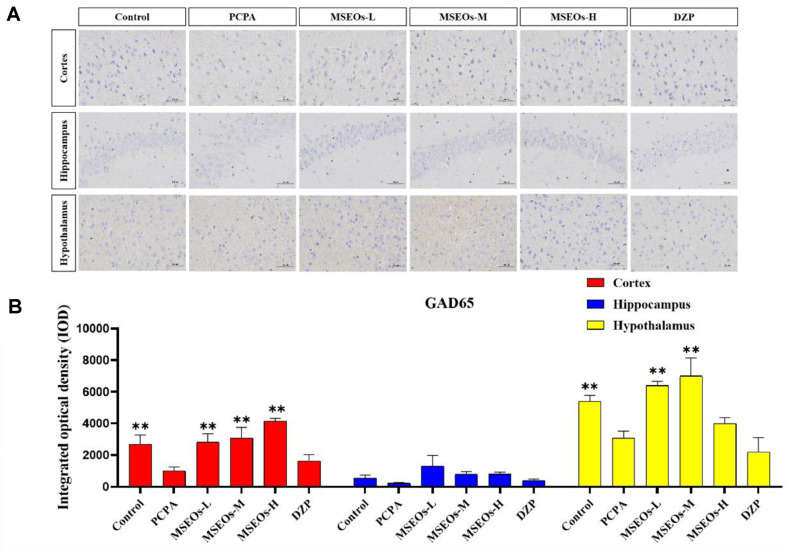
Expression of GAD65 protein. (**A**) Images of GAD65 protein staining in various regions of mouse brain tissue; (**B**) The combined optical density across various brain tissue sections was assessed. (*p < 0.05 and **P < 0.01 highlights notable distinctions from the PCPA-induced model group. MSEOs-L, MSEOs-M, and MSEOs-H represent dosages of 25 mg/kg, 50 mg/kg, and 100 mg/kg, respectively.) Data showed mean ± SD (*n* = 4).

The GAD65 content in the cortex and hypothalamus of the PCPA group was markedly lower than that of the control group (*P* < 0.01) (Fig. 8B), indicating that intraperitoneal injection of PCPA results in a reduction of GAD65 levels within the brain tissue. In contrast, GAD65 levels in the hypothalamus, cortex, and hippocampus of mice administered with MSEOs were elevated compared to both the blank and the PCPA groups, In the study, the MSEOs-H group showed the most significant effect in the cerebral cortex (*P* < 0.01), while the MSEOs-L group performed better in the hippocampus, and the MSEOs-M group was most prominent in the hypothalamus (*P* < 0.01). The result indicates that treatment with MSEOs is capable of effectively preventing the decline in GAD65 protein levels in the cerebral tissue of mice experiencing PCPA-induced sleep disturbance, maintaining the GAD65 content in their bodies at normal levels.

### RT- qPCR

**Fig. 9:**
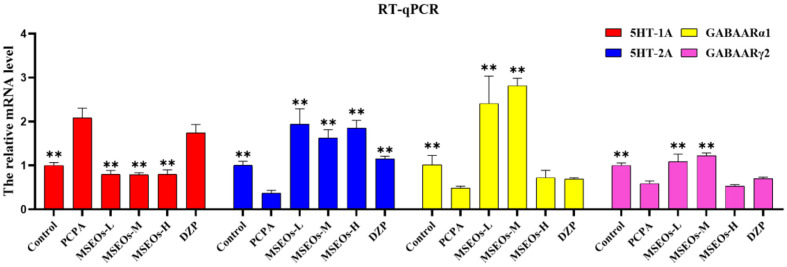
The comparative expression levels of mRNA for GABAARα1, GABAARγ2, 5HT-2A, and 5HT-1A. (**P < 0.01, *P < 0.05 highlight notable distinctions from the PCPA-induced model group. MSEOs-L, MSEOs-M, and MSEOs- H represent dosages of 25 mg/kg, 50 mg/kg, and 100 mg/kg, respectively.) Data showed mean ± SD (*n* = 4).

The results of RT-qPCR (Fig. 9) indicated that relative to the control, the mRNA expression of 5HT-2A, GABAARα1, and GABAARγ2 in the insomnia PCPA-treated mice was diminished. Following treatment with MSEOs, the mRNA levels of GABAARα1, GABAARγ2, and 5HT-2A in brain tissue of the three different concentrations of MSEOs treatment groups showed varying degrees of upregulation when compared to the insomnia model group. The expression of 5HT-2A was also significantly increased after MSEOs-H treatment (*p* < 0.01), followed by the MSEOs-L treatment group (*p* < 0.01), showing consistency with the staining results of 5HT-2A in the cerebral cortex, hypothalamus, and hippocampus. For the expression of GABAARα1, the enhancement effect was most pronounced in the MSEOs-M treatment group (*p* < 0.01), followed by the MSEOs-H treatment group (*p* < 0.01), which was roughly similar to the staining pattern of GABAARα1 in the corresponding brain regions. As subunits of GABAAR, α1, and γ2 subunits are interdependent in receptor function, and the increase in GABAARγ2 also contributes to the treatment of insomnia [42]. The results in Fig. 9 show that the expression patterns of GABAARα1 and GABAARγ2 were consistent, with the most significant increase after MSEOs-M treatment (*p* < 0.01), followed by the MSEOs-L treatment group (*p* < 0.01). These findings provide strong molecular biological evidence for the effectiveness of MSEOs in the treatment of insomnia.

## Discussion

In recent years, the potential of MSEOs in treating insomnia has gradually become evident [12]. The results of this study indicate that MSEOs can improve the symptoms of insomnia induced by PCPA in mice and regulate the levels of related neurotransmitters. Compared to chemical drugs, MSEOs demonstrate mild efficacy and possibly a lower risk of adverse reactions, providing a new treatment option for patients with insomnia. Behavioral experiment results show that MSEOs can significantly shorten the latency to sleep and extend the total sleep time in mice, indicating a positive regulatory effect on the sleep-wake cycle. This effect may be related to the ability of MSEOs to regulate neurotransmitter levels through the hypothalamus, particularly the regulation of 5HT-1A, 5HT-2A, GABAARα1, and GAD65. This study expands the understanding of the mechanism of action of MSEOs in the existing literature, not only verifying their impact on 5HT-1A and GABAARa, but also analyzing the expression levels of 5HT-2A, GAD65, and GABAARγ2. In this study, through the analysis of MSEOs components, we identified the main components as β-elemene, (Z)-β-ocimene, Germacrene D, cis-(+)Nerolidol, and isocaryophyllene. Previous studies have confirmed that β-elemene and cis-(+)Nerolidol possess anti-neuroinflammatory properties, and cis-(+)Nerolidol also has a sedative effect. These anti-inflammatory effects are closely related to the GABAergic system, and these components can enhance neuronal function by activating 5-HT receptors, particularly the 5HT-1A and 5HT-4 subtypes. Given the key role of the GABAergic system and 5-HT receptors in the pathological mechanism of insomnia [23, 46], we found that the MSEOs containing these components significantly improved the reduced expression of 5-HT1A and GABAARα1 in a mouse model of insomnia. Based on this, we speculate that β-elemene and cis-(+)Nerolidol may be the crucial components in the MSEOs for their anti-insomnia effects and demonstrate potential for the treatment of insomnia. Future research directions will focus on validating the therapeutic effects of these individual components on insomnia, as well as exploring the potential application of these compounds in new drug development and compound formulations. These results provide a more in-depth experimental basis for the application of MSEOs in the field of insomnia treatment. In this study, the three concentration gradients of MSEOs (low, medium, and high) all showed positive therapeutic effects on various indicators, but there was some inconsistency in efficacy. This difference in efficacy may be attributed to the instability of essential oils [47] and the possible subtle differences in the daily inhalation dose of the drug in mice during the one-week continuous administration period. Nevertheless, the overall effect of MSEOs against insomnia is still significant. Therefore, future research can focus on developing different formulations of MSEOs, such as using liposomes to encapsulate essential oils, to overcome their stability issues, which would be beneficial for clinical application. In summary, MSEOs, as a natural plant product, have the potential to become a natural alternative therapy for the treatment of insomnia, providing a reliable treatment plan based on traditional Chinese medicine for clinical use.

## Conclusion

The results of this study indicated that MSEOs could improve insomnia by prolonging the total sleep time and reducing sleep latency in mice. Furthermore, MSEOs can modulate key targets in the sleep-wake regulation pathway, as evidenced by increased levels of GAD65, GABAARα1, 5HT-2A, and 5HT-1A following treatment. Additionally, MSEOs were found to mitigate PCPA-induced neuronal damage, suggesting a neuroprotective role. Indicating that MSEOs may act by enhancing the GABAergic and serotonergic systems to elicit their sedative and neuroprotective effects. These findings underscore the potential of MSEOs as a therapeutic agent for sleep disorders and as a neuroprotective agent against neurodegenerative conditions.

## Figures and Tables

**Table 1:** The name, voucher specimen number, and storage location of the plant sample.

Latin name	Local name	Voucher number	Collection time	Storage location
*Magnolia sieboldii*	Tiannvmulan	2020-112A	2020.09	Institute of Natural Medicine & Green Chemistry, School of Biomedical and Pharmaceutical Sciences, Guangdong University of Technology

**Table 2:** Primer sequences information.

Genes	Forward (5'-3')	Reverse (3'-5')
5HT-1A	CCAACTATCTCATCGGCTCCTT	CTGACCCAGAGTCCACTTGTTG
5HT-2A	TATGCTGCTGGGTTTCCTTGT	GTTGAAGCGGCTATGGTGAAT
GABAARα1	ATGACAGTGCTCCGGCTAAAC	AGTGCATTGGGCATTCAGCT
GABAARγ2	GCAGTTCTGTTGAAGTGGGTGA	GCAGGGAATGTAAGTCTGGATGG

**Table 3:** Relative content (%) and retention index (RI) of each compound identified from *Magnolia sieboldii* essential oils.